# Bacterial Communities Associated with Houseflies (*Musca domestica* L.) Sampled within and between Farms

**DOI:** 10.1371/journal.pone.0169753

**Published:** 2017-01-12

**Authors:** Simon Bahrndorff, Nadieh de Jonge, Henrik Skovgård, Jeppe Lund Nielsen

**Affiliations:** 1 Department of Chemistry and Bioscience, Aalborg University, Aalborg East, Denmark; 2 Department of Agroecology, University of Aarhus, Slagelse, Denmark; University of Illinois at Urbana-Champaign, UNITED STATES

## Abstract

The housefly feeds and reproduces in animal manure and decaying organic substances and thus lives in intimate association with various microorganisms including human pathogens. In order to understand the variation and association between bacteria and the housefly, we used 16S rRNA gene amplicon sequencing to describe bacterial communities of 90 individual houseflies collected within and between ten dairy farms in Denmark. Analysis of gene sequences showed that the most abundant classes of bacteria found across all sites included Bacilli, Clostridia, Actinobacteria, Flavobacteria, and all classes of Proteobacteria and at the genus level the most abundant genera included *Corynebacterium*, *Lactobacillus*, *Staphylococcus*, *Vagococcus*, *Weissella*, *Lactococcus*, and *Aerococcus*. Comparison of the microbiota of houseflies revealed a highly diverse microbiota compared to other insect species and with most variation in species richness and diversity found between individuals, but not locations. Our study is the first in-depth amplicon sequencing study of the housefly microbiota, and collectively shows that the microbiota of single houseflies is highly diverse and differs between individuals likely to reflect the lifestyle of the housefly. We suggest that these results should be taken into account when addressing the transmission of pathogens by the housefly and assessing the vector competence variation under natural conditions.

## Introduction

The microbiota of insects can contribute to various aspects of host physiology, such as nutritional supplementation, tolerance to environmental perturbations, providing colonization resistance against pathogenic organisms, and priming the development and maturation of the host immune system [1,2]. It has thus been suggested that variation in the microbiota between individuals can explain differences in the phenotype, such as vector competency of vectors. However, few studies have addressed variation in time and space of the microbiota between individuals under field conditions.

The ability of bacteria to colonize and persist within an insect is dependent on the insect immune system [3]. For example, different pathogens can induce the innate immune response of the host affecting the retention time of the pathogen [4]. The genetics of the host may also affect the ability of bacteria to persist in the insect and thus the vector competence of the host [5,6]. Recently, the role of the microbiota of insect vectors for the transmission of human pathogens has received attention [7–9] and the microbiota of many insects known to be vectors of human pathogens have been described, especially for blood-feeding insects [10–14]. Results indicate that in several insect systems, direct and indirect microbiota induced phenotypes can affect the capacity of insect vectors to transmit human pathogens and thus impact the host vector competence [9,15]. It has thus been proposed that the ability of some pathogenic bacteria to colonize and persist within an insect is not only dependent on the insect immune system and genetics, but also the existing microbiota [10]. Similarly, some insects are vectors of bacterial diseases including human pathogens [16,17], whereas other species, such as mosquitoes, are not known to harbor pathogenic bacteria. More microbiota studies on non-blood-feeding insects are therefore needed to establish if the microbiota can help explain these differences.

Studies have also suggested that abiotic factors can affect the microbiota of disease vectors and thus vector competence of the host [10,11], which might explain some of the seasonal variation in epidemics of human pathogens. This is in agreement with results showing that the epidemics of human pathogens transmitted by insect vectors often correlate with environmental factors [16] and that the vector competence of insect vectors is affected either indirectly or directly by environmental factors [4,10,18].

Many synanthropic flies, including blood-feeding species, live in close association with bacteria and breed and feed in habitats, such as animal manure, human excrement, garbage, animal bedding, or decaying organic matter rich in microorganisms [19]. Both the feeding mechanisms and breeding behavior of synanthropic flies make them efficient biological or mechanical vectors of human pathogens. Especially muscoid flies, such as the housefly, *Musca domestica* (Diptera: Muscidae), are known as carriers of many disease causing microorganisms including bacteria, virus, fungi, and parasites [19]. Results have shown the housefly to be an effective vector of pathogens, such as *Campylobacter* spp. and *Shigella* spp. [16,17], and carrier (carrier if no bacterial multiplication occurs) of bacteria, such as *Campylobacter jejuni* [20], *Salmonella* spp. [21], *Shigella* spp. [17], *Staphylococcus aureus* [22], *Pseudomonas aeruginosa* [22], *Enterococcus faecalis* [22], and *Escherichia coli* [23]. High activity and dispersal potential results in increased fly contamination and transmission through fecal deposits and/or extracorporeal digestion [19,24]. Bacteria also play a significant role for the successful development of larvae of the housefly [25], and results show that larvae of the housefly fail to grow in an axenic environment [26]. This is not surprising given that all life stages of houseflies (egg, larvae, pupae, and adults) are in contact with various microorganisms. Symbiotic associations of microorganisms with the housefly have also been found to affect the oviposition behavior [27].

The close association of the housefly and bacteria, and its role in transmission of pathogens, makes it an ideal model organism to study the importance and variation of the microbiota of vector species. Few studies have addressed the variation in the microbiota of filth flies under natural conditions although such variation is likely to affect the phenotype of the fly. In order to explore the bacterial communities associated with the housefly, and to identify the variation found under natural conditions, we surveyed natural populations of *M*. *domestica* collected at 10 dairy farms throughout Denmark. We used culture-independent amplicon sequencing of the 16S rRNA gene to characterize the bacterial communities and richness associated with individual houseflies and across locations. The approach being used allowed higher resolution compared to earlier studies addressing the microbiota of houseflies [28–30], and provide the first comprehensive survey of the entire microbiota associated with a major synanthropic vector of pathogenic bacteria. Individual flies were used to assess variation in the bacterial communities within and between farms and multiple flies were collected at each farm. These data will provide an important step in understanding the variation of host-microbe interactions in an important vector of human pathogens under field conditions.

## Materials and Methods

### Ethical statement

The specimens used in this study were collected at farms owned by private farmers. Housefly collections were done with the approval of the farmers. No endangered or protected species were included in the present study.

### Samples

**Fig 1:**
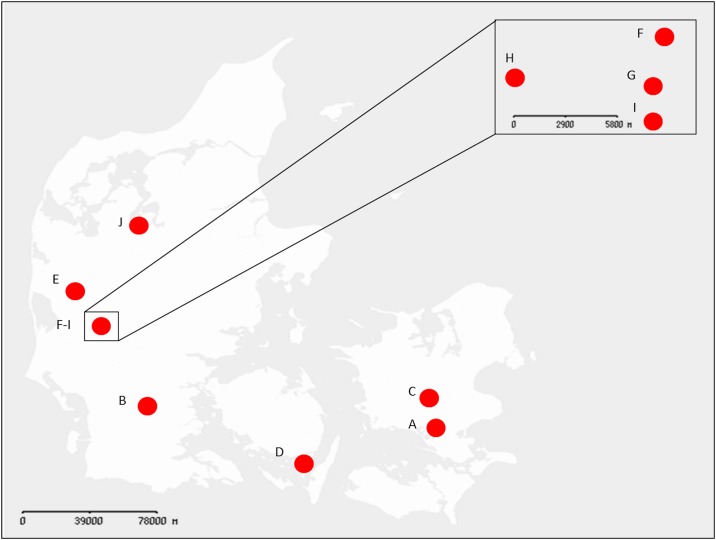
Map of locations from where flies were collected. From each location (red circles) the microbiota of 7–10 male flies were established (A = 8; B = 7; C = 8; D = 10; E = 9; F = 9; G = 9; H = 10; I = 10; J = 10). Locations are designated with a letter (A-J). DNA was extracted from whole flies. In total 10 locations (dairy farms) were sampled throughout Denmark, where 4 locations (F-I) were in close proximity of each other (< 10 km). A GeoDanmark Basis map was downloaded from the Danish “Geodatastyrelsen”, October 2016, Styrelsen for Dataforsyning og Effektivisering. The figure is similar, but not identical to the original image, and is therefore for illustrative purposes only.

Flies were collected in late summer during a 24 hour period (6-7^th^ of September in 2012) from 10 dairy farms using a sweeping net (Fig 1). Flies were collected from the inside of the farms in closed areas with calves walking on deep litter. The farms were located throughout Denmark and were all farms with similar farming practice and manure management (A, Næstved, 55.11N, 11.47E; B, Rødding, 55.20N, 09.10E; C, Ringsted, 55.23N, 11.47E; D, Svendborg, 54.58N, 10.38E; E, Spjald, 56.06N, 08.30E; F, Sdr. Felding, 55.54N, 08.47E; G, Tarm, 55.53N, 08.43E, H, Tarm, 55.52N, 08.39E; I, Tarm, 55.51N, 08.46E; J, Højslev, 56.32N, 09.07E). Flies were immediately stored in 99.5% ethanol and kept on ice upon transport to the laboratory and subsequently stored at -20°C until further processing. In the laboratory flies were allocated into individual sterile vials and handled with a pair of sterile forceps to minimize contamination between flies. *Musca domestica* species identity of flies was established as described elsewhere [31]. Only male flies were investigated to avoid any potential sex-dependent variations, and thus enable testing a higher number of individuals per site. Large differences between the sexes affecting dispersal range and activity of houseflies have been reported elsewhere [24,32], and these could potentially influence the composition of the microbiota.

### DNA extraction and 16S rRNA gene amplicon sequencing

Total DNA of whole male flies was extracted using the DNeasy^®^ Blood & Tissue Kit (Qiagen, Inc., Hilden, Germany) and following the Qiagen supplementary protocol for purification of total DNA from insects. Flies were grinded in liquid nitrogen using a pestle in 1.5 ml microcentrifuge tubes. Subsequently 180 μl ATL buffer and 20 μl of proteinase K were added and incubated overnight at 56°C. The day after 4 μl of RNase (100mg/ml) was added to each sample and incubated for 1 hour at room temperature before following the standard protocol. DNA from each sample was eluted into 100 μl of AE-buffer and subsequently stored at -20°C until further use. DNA quantity and quality was verified using a fluorometer (Qubit^®^, Thermo Fisher Scientific Inc.) and by using gel electrophoresis with a 1% (w/v) agarose gel.

In order to determine the microbiota associated with the housefly, we amplified and sequenced a part of the 16S rRNA gene spanning the variable regions V1, V2 and V3. The V1-3 hypervariable region of the bacterial 16S rRNA gene was amplified using the V1-3 primers 27F AGAGTTTGATCCTGGCTCAG and 534R ATTACCGCGGCTGCTGG identical to the primers used by Human microbiome project [33]. The samples were sequenced in equimolar concentrations on a MiSeq (Illumina, USA) using MiSeq reagent kit v3 (2x300 PE).

### Bioinformatic processing and statistical analysis

The obtained sequence libraries were trimmed and their low quality reads removed using trimmomatic (v0.32) [34]. Reads were merged using FLASH (v1.2.7) [35]. Chimera were removed and reads were then formatted for use with the UPARSE workflow [36]. Usearch7 was used to de-replicate reads and cluster them into Operational Taxonomic Units (OTUs) at 97% similarity. Taxonomy was assigned using RDP classifier [37] as implemented in QIIME [38], using GreenGenes as a reference database [39].

The statistical analyses and visualizations were performed in R version 3.2 (R core team, 2015) via RStudio version 0.99 (http://www.rstudio.com), using the R packages phyloseq [40], vegan, ampvis [41] and ggplot2 [42]. Biodiversity was explored using alpha diversity indices, such as Chao1, Shannon and Simpson [43–45]. Diversity indices did not meet the assumptions of equal variances and locations were therefore compared using a nonparametric Kruskal—Wallis test, which tests for differences between the medians of the distribution and pairwise comparisons using Dunnett tests [46]. Beta diversity was calculated for microbiota comparison between *M*. *domestica* from different locations using Bray-Curtis dissimilarity [47] or UniFrac metrics [48], using only OTUs with an abundant presence (>0.1% of total reads in at least 1 sample). Principal coordinate analysis (PCoA) was used to visualize differences between microbial communities and the microbial community structure using heatmaps. A phylogenetic tree of 16S rRNA gene sequences was generated using the Unweighted Pair Group Method with Arithmetic Mean (UPGMA) clustering algorithm with 1,000 bootstrap replications to explore relationships of abundant OTUs within the families of Campylobacteraceae, Enterobacteriaceae, Staphylococcaceae, Enterococcaceae, and Pseudomonadaceae. These families were chosen as they are known to harbor potential pathogens carried by houseflies and were used as examples to illustrate the variation found within and between locations. All amplicon data are available at European Nucleotide Archive (ENA) under project number PRJEB15078 (http://www.ebi.ac.uk/ena/data/view/PRJEB15078).

## Results

The combined amount of DNA from the fly and the microbiota extracted was 2,324±983 ng DNA per fly (mean±SD) and the 16S rRNA gene amplicon sequencing yielded 2,075,663 sequences with an average number of 16,563±6,989 per sample. All sequences were subsequently grouped into operational taxonomic units (OTUs) at 97% sequence similarity. Only samples for which a total number of 5,000 reads or more were collected were considered for analyses.

### Diversity of bacterial communities within and between locations

**Fig 2:**
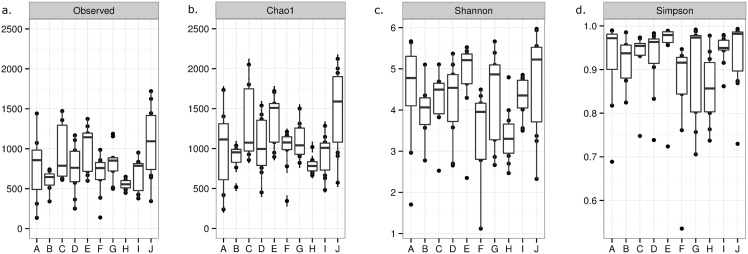
Diversity measurements of the bacterial communities of houseflies sampled across location. Boxplot displaying the observed number of OTUs (a), richness (b; Chao1), evenness (c; Shannon’s index) and biodiversity (d; Simpson’s index) per location (A-J) of sampling (n = 7–10). The boxplot bounds the interquartile range (IQR) divided by the median, the whiskers extend to 1.5x IQR beyond the box.

OTU richness, evenness, and overall diversity of bacteria varied widely among flies within locations and among locations (Fig 2). An average number of 749±330 OTUs were observed across locations, with location J showing the highest number (1,092±450) of OTUs identified (Fig 2a). Species richness between all samples was visualized by rarefaction curve (S1 Fig) in order to assess sequencing depth. The curves level off after 5,000 sequences per sample (the minimum number of reads we determined to be acceptable per sample), which indicates that sequencing depth was sufficient for analysis. The plot also underlines the variation seen between samples and sampling locations in terms of richness (alpha diversity). Calculated species richness (Chao1) was significantly different between locations (H = 24.23; df = 9; p = 0.004) (Fig 2b) and with a projected diversity that exceeds the observed diversity by an average of 27%. The Chao1 value for population H was significantly lower compared to population J and E (p<0.05). As for the evenness (Shannon entropy), all locations showed a Shannon diversity ranging between 3–5 and was significantly different between locations (H = 20.02; df = 9; p = 0.018) (Fig 2c), which is indicative of a diverse and complex community, without many dominating organisms. The Shannon diversity index value for population H was significantly lower compared to population E (p<0.05). The high bacterial diversity found within and across locations is supported by a high Simpson index (Fig 2d), where almost all locations in the present study showed an average Simpson index of more than 0.9, suggesting that the houseflies present a very diverse habitat in terms of the microbiota. The Simpson index differed significantly between population (H = 19.79; df = 9; p = 0.019).

**Fig 3:**
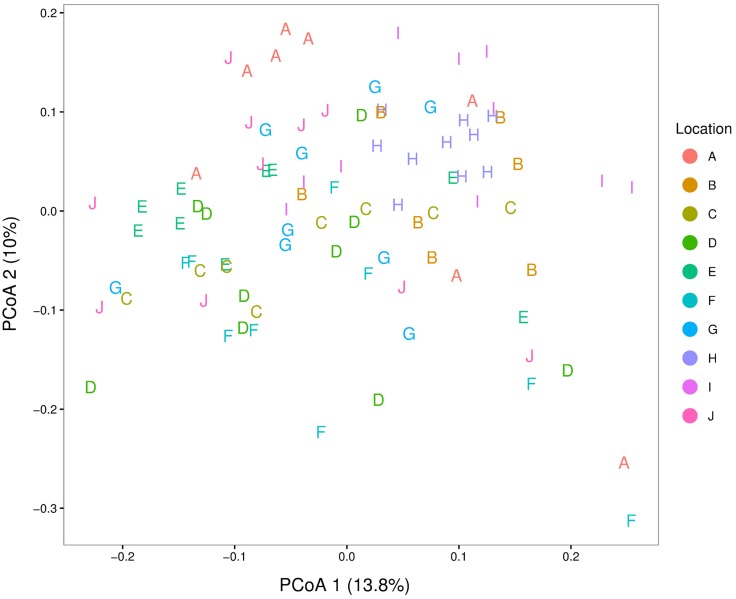
Principal coordinate analysis (PCoA) of the housefly microbiota. PCoA of unweighted UniFrac distances between samples. Samples are colored by location and designated as A-J. OTUs with an abundant (>0.1% of total reads in at least 1 sample) presence were included in the analysis.

We found a significant effect of location (adonis test, p = 0.001, R^2^ = 0.22), when using the Bray-Curtis dissimilarity matrix. However, the beta diversity differences are small and when performing principal coordinate analysis (PCoA) on unweighted UniFrac measures the locations are overlapping (Fig 3). The first ordinate (PCoA1) explains 13.8% of the variation, whereas the second ordinate (PCoA2) explains 10%.

### Bacterial community composition

**Fig 4:**
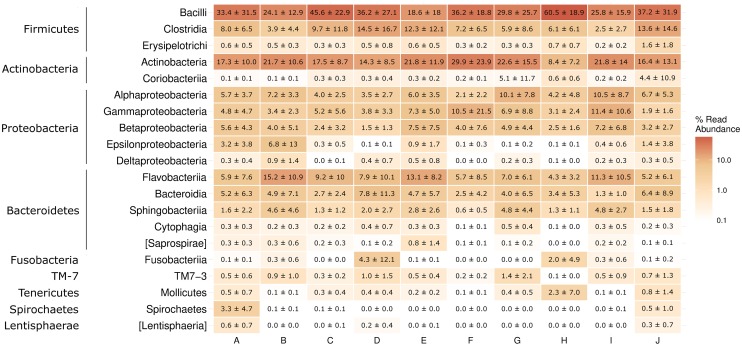
Composition and abundance of the housefly microbiota. Heatmap (mean ± SD) representing the 20 bacterial classes (sorted by phylum) with the highest relative abundance. Samples are sorted by location.

**Fig 5:**
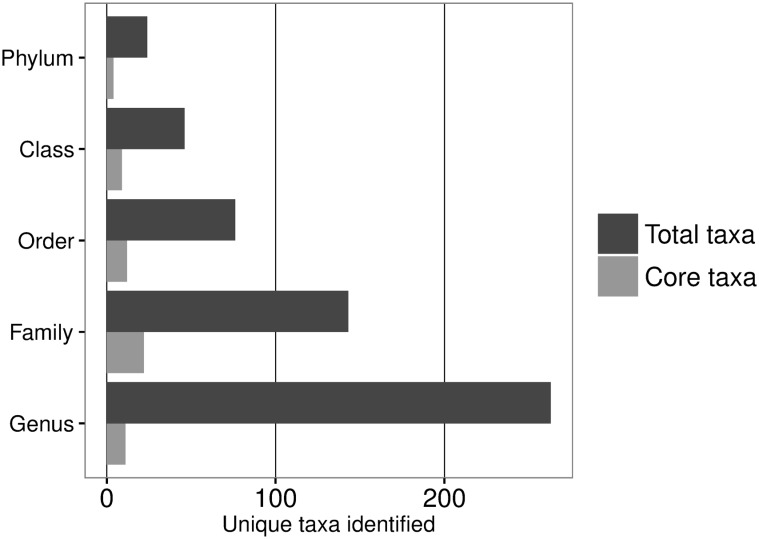
Barplot displaying number of abundant and core taxa of the housefly microbiota per taxonomic level. The total number of abundant taxa was defined as >0.1% abundance in at least 1 sample (dark grey) and core taxa as occurring and abundant in 80% of all samples (grey). OTUs with an abundant (>0.1% of total reads in at least 1 sample) presence were included in the analysis.

**Fig 6:**
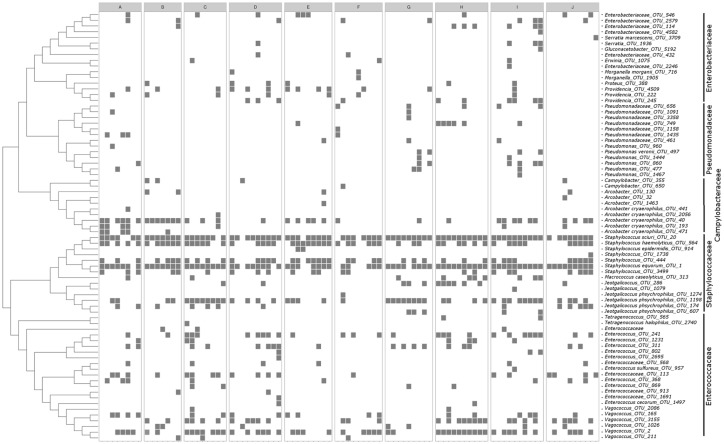
Phylogenetic tree and heatmap of selected families. Heatmap displaying the abundant occurrence (present/non present) of the OTUs identified from each family, Enterobacteriaceae, Pseudomonadaceae, Campylobacteraceae, Staphylococcaceae, and Enterococcaceae. Samples are ordered via a UPGMA based phylogenetic tree based on their 16S rRNA gene consensus sequences. Some OTUs were only identified to family (f = family). OTUs with an abundant (>0.1% of total reads in at least 1 sample) presence were included in the analysis.

The bacterial taxa associated with the housefly across sites showed that the microbiota of houseflies is dominated by the phyla Firmicutes, Actinobacteria, Proteobacteria, and Bacteroidetes (>92% of total reads) (S2 Fig). Smaller contributions were made by Fusobacteria, TM7, and Tenericutes (2% each). Locations B, E, G, and I showed an even distribution between the four most abundant phyla, whereas for population A, C, D, F, and J up to 50% of the total reads were represented by Firmicutes and the other 3 large phyla, contributing on average 15% of the total reads. Location H differed slightly from the other population, where bacteria from the phyla Firmicutes dominated the microbiota (67.3% of the total reads). Some of the most abundant classes of bacteria found across sites included Bacilli, Clostridia, Actinobacteria, and all classes of Proteobacteria and some of the minor classes present across all sites included Bacteroidia, Erysipelotrichi, Sphingobacteria, Cytophagia, Coriobacteriia, TM7, and Mollicutes (Fig 4). Again population H showed slightly different composition compared to the other locations with 60.5% of the total reads affiliating with a single class (Bacilli). For the remaining locations the classes Bacilli and Actinobacteria were the most abundant, but with large differences for the remaining classes. Similar patterns were seen at the genus level with large variation of which organisms that were the most abundant at each location (S3 Fig). The number of unique OTUs assigned to a given taxonomic level thus also increased with taxonomic resolution (Fig 5). However, OTUs that are both abundant and occur in more than 80% of all samples showed the highest number at the family level. In total, 411 unique OTUs were identified at the genus level and of these 263 were classified as abundant. The OTUs contained bacteria or groups of bacteria, such as *Enterococcus*, *Campylobacter*, *Pseudomonas*, and *Klebsiella*. For example, *Campylobacter* spp. was present in 38 flies out of 90, but only classified as abundant in 5 flies (Fig 5). Phylogenetic analysis of 16S rRNA gene sequences from the family Campylobacteraceae showed that most OTUs grouped into the genera *Arcobacter* or *Campylobacter* (Fig 6). For Enterococcaceae, gene sequences were identified as belonging to the genera *Tetragenococcus*, *Enterococcus*, or *Vagococcus* and for Enterobacteriaceae belonging to *Morganella*, *Providencia*, *Proteus*, *Erwinia*, *Serratia*, *Gluconacetobacter*, and *Serratia*. Some sequences were only identified to family level. For Pseudomonadaceae, sequences were identified as belonging to the family Pseudomonadaceae or the genus *Pseudomonas*. For Staphylococcaceae, sequences were identified as belonging to the genera *Jeotgalicoccus*, *Macrococcus*, and *Staphylococcus*. The well-known endosymbiotic bacterium of many insects, *Wolbachia*, was found in 4 flies, but all from different locations.

## Discussion

The microbiota of insects play an important role in both host nutritional physiology and immune competence and can thus affect the fitness of insect vectors [9]. However, few studies have addressed the variation of the microbiota of insects under field conditions. Further, most studies on the microbiota of insect vectors have focused on blood-feeding species of mosquitoes, tsetse flies and fleas [10,12,14,49] and generally with limited spatial resolution and carried out by pooling of host samples. Fewer studies have addressed the microbiota of non-biting insect vectors, such as filth flies and often with limited taxonomic resolution of host species [11,13,28,30].

The present study provides an in-depth analysis of the adult housefly microbiota and unlike other microbiota studies of filth flies [11,13,28,50,51], we looked at the microbiota of single whole flies sampled within and between locations to insure that we included the entire microbiota of single flies and variation under natural conditions. The results provide a culture-independent description of the microbiota of a vector of public health importance and show a large and highly diverse microbiota compared to other insect vectors [11,52,53].

Studies have shown large variations in vector competence of different arthropod species [54,55] and individuals [56] and suggested that this variation could be explained by variation in the microbiota [8,9]. However, few studies have in fact addressed the variation in the microbiota of insects found within and between populations or locations under natural conditions. The results of the present study certainly suggest that the microbiota of the housefly is more diverse compared to other species and with variation in the microbiota within and between locations. Our findings support studies on populations of the tsetse fly and fleas [10,14], and indicate the presence of a very small core microbiota and large variations in the microbiota between individuals. These observations are important to consider when establishing the vector competence of bacteria for insect species as large variations might exist under natural conditions. The large differences between the microbiota of individual houseflies within sampling sites are somewhat surprising, given the promiscuous nature of houseflies, which could increase transmission rates between individuals [57]. One explanation for this might be due to the lower abundance and diversity of the ectomicrobiota compared to the endomicrobiota, although this needs to be clarified for houseflies [58]. Cross contamination during collection of flies could also affect the variation between flies. We would expect that any cross contamination would reduce the variation between individuals and lead to an underestimation of the actual variability.

In this study we have investigated the entire microbiota of the housefly and not only restricted the analysis to the gut microbiota. We hypothesize that transmission of microorganisms by the housefly is better reflected through the investigation of the entire microbiota. Some of the major genera identified in the present study are similar to previous studies looking at the microbiota of synanthropic flies with both culture-dependent and -independent methods. Other studies have applied culture-dependent and culture-independent methods to assess the microbiota of pooled housefly samples collected from diverse habitats, such as a garden, restaurant, mutton shop and human houses and found a high abundance of bacteria belonging to *Staphylococcus*, *Vagococcus*, *Wohlfahrtiimonas*, or *Ignatzschineria* [28]. Similarly, dominant genera of the microbiota of the green bottle fly species included *Lactobacillus*, *Staphylococcus*, *Vagococcus*, and *Acetobacter* [11] and *Weissella*, *Wohlfahrtiimonas*, and *Ignatzschineria* of flesh fly species [13]. Many of the genera found in the present study have not been found associated with the housefly before. Alpha diversity measures were also higher in the present study compared to other studies looking at the microbiota of arthropods [52,53,59–61]. Average Chao1 ranged from 750 to 1,500 and Shannon diversity index from 3.4 to 5.4. Results obtained on the housefly using cloning based approaches showed much lower values [11,13,28], whereas amplicon sequencing to address the microbiota of the green bottle fly revealed similar values for diversity (Shannon and Simpson) [11]. Collectively these results suggest that synanthropic flies, and especially the housefly, show a high species diversity and richness in the bacterial community. The high species richness of the housefly microbiota is likely to reflect its lifestyle breeding and living by animal manure, bedding, and decaying organic matter rich in microorganisms [19,62].

It is well documented that the diet of the host can play a major role in shaping the microbiota [60,63] and this might be especially true for the housefly due to its feeding biology, exposing individuals to a diverse array of bacteria [62]. We collected flies only from dairy farms in the present study and it could thus be expected that the housefly microbiota would reflect this habitat. Earlier studies have identified major classes of bacteria found in the rumen content of cows, which were also found in houseflies in the present study and included Clostridia, Erysipelotrichi, Bacilli, Spirochaetes, Betaproteobacteria, Gammaproteobacteria, Alphaproteobacteria, Mollicutes, Bacteroidetes, and Actinobacteria [64]. However, at the genus level significant differences exists and among the major genera only *Bacteroides* and *Streptococcus* were similar [64,65]. Fig 5 shows the limited overlap of the core taxa which occurs in at least 80% of all samples at different taxonomic levels. Farms may provide different niches and types of food sources available for houseflies, which could explain the highdiversity and variation in the housefly microbiota. Future studies are needed to clarify the importance of habitats in shaping houseflies’ microbiota and linking microbial communities with the environment. Dispersal and activity of houseflies might further complicate the link between habitat and the housefly microbiota. Houseflies are able to fly long distances and flies collected at one farm might originate from close by farms with different farming practices [24,66].

In the present study we were able to identify multiple species of bacteria to the genus and species level. It is evident that some genera or species, potentially harboring human pathogens, are only present at single flies or farms, whereas other species or genera are present across all sites and on most flies (Fig 6). Genera of specific interest that might harbor human pathogens included *Enterococcus*, *Staphylococcus*, *Pseudomonas*, *Campylobacter*, and *Klebsiella*. Of these, the genus *Campylobacter* is of special interests. *Campylobacter* spp. is recognized as one of the leading bacterial causes of gastroenteritis in the world and with campylobacteriosis largely perceived to be a food borne disease [67]. The house fly is a well-established vector of *Campylobacter* spp. and can cause infection of broiler chicken flocks, and through contaminated broiler meat can cause outbreaks of campylobacteriosis in humans [16,67]. It has been estimated, by applying culture dependent approaches, that the prevalence of *Campylobacter* spp. positive flies varies from 0 to 16% on broiler farms [68]. In our study, using culture independent approaches, we found that 42% of all houseflies were found to contain *Campylobacter* spp. highlighting the importance of the housefly as a vector of *Campylobacter*. The bacterial symbiont, *Wolbachia* spp., was present in less than 4% of the flies of the present study. However, the investigated gene used in the present study does not provide information on the pathogenicity. This intracellular symbiotic are found in an estimated 20–70% of insect species [69]. Studies have found that *Wolbachia* can affect the ability of insects to transmit pathogens either indirectly through reduced lifespan [70] or directly by reducing the ability of pathogens to proliferate within the insect, both in the laboratory and in the field [71,72]. However, the ecological role and significance of these bacteria in the housefly remains unclear.

The differences in the microbiota between individual flies are relevant for epidemiological studies predicting the distribution of hosts carrying pathogens. The results of the present study show large variation between individuals and suggest that the number of flies sequenced is important for understanding dynamics under natural conditions. The diversity found among the general microbial communities in this study suggests that limited sampling or pooling of individual houseflies for estimating the transfer of pathogens might not be representative of fly populations under natural conditions. Experimental and sampling designs should therefore take into account local and regional differences in the microbiota of houseflies. With the setup of the current study we were able to evaluate the variation found within and between populations, but not establish what factors that determine the microbiota of the flies. Our results suggest that large differences can exist under natural conditions, which could indicate that the variation present in laboratory cultures will not reflect variation found under natural conditions.

## Conclusions

The microbiota of arthropods plays an important role in host nutrition and can influence the transmission of vector borne pathogens. In the present study we have for the first time examined the microbiota of adult *Musca domestica* from different geographical farms with similar farming practice and manure management. Using 16S rRNA gene amplicon sequencing of individual flies and on the entire fly microbiota revealed a highly diverse microbiota compared to other arthropods. Interestingly, the approach of investigating individual flies shows that most variation was found between individuals within locations and with smaller differences between locations for both richness and diversity. We recommend that the large variation in the housefly microbiota found under natural conditions should be taken into consideration when trying to establish the vector competence of bacteria for insect species, but also in epidemiological studies addressing the spread and distribution of pathogens by vector species.

## Supporting Information

S1 Fig Rarefaction curves of OTUs clustered at 97% similarity for the housefly individuals collected at 10 farms. (EPS)

S2 Fig Heatmap (mean ± STD) representing the bacterial phyla found, samples are sorted by location. (EPS)

S3 Fig Heatmap (mean ± STD) representing the 25 most abundant bacterial genera, samples are sorted by location. Some OTUs were only identified to order or family (o = order and f = family).
(EPS)
